# Global Expression Profiling Identifies a Novel Hyaluronan Synthases 2 Gene in the Pathogenesis of Lower Extremity Varicose Veins

**DOI:** 10.3390/jcm7120537

**Published:** 2018-12-11

**Authors:** Chia-Shan Hsieh, Chia-Ti Tsai, Yau-Hung Chen, Sheng-Nan Chang, Juey-Jen Hwang, Eric Y. Chuang, I-Hui Wu

**Affiliations:** 1 Department of Life Science, Genome and Systems Biology Degree Program, National Taiwan University, Taipei 10617, Taiwan; cometrise@gmail.com; 2 Bioinformatics and Biostatistics Core, Center of Genomic Medicine, National Taiwan University, Taipei 10055, Taiwan; 3 Division of Cardiology, Department of Internal Medicine, National Taiwan University College of Medicine and Hospital, Taipei 10002, Taiwan; cttsai1999@gmail.com (C.-T.T.); jueyhwang@ntu.edu.tw (J.-J.H.); 4 Graduate Institute of Clinical Medicine, College of Medicine, National Taiwan University, Taipei 10051, Taiwan; 5 Department of Chemistry, Tamkang University, Taipei 25137, Taiwan; yauhung@mail.tku.edu.tw; 6 Department of Internal Medicine, National Taiwan University Hospital Yun-Lin Branch, Yun-Lin 64041, Taiwan; p95421008@ntu.edu.tw; 7 Department of Surgery, National Taiwan University Hospital, Taipei, 10002, Taiwan

**Keywords:** varicose veins, *HAS2*, RNA seq

## Abstract

Lower extremities varicose veins (VV) are among the most easily recognized venous abnormalities. The genetic mechanism of VV is largely unknown. In this study, we sought to explore the global expressional change of VV and identify novel genes that might play a role in VV. We used next-generation ribonucleic acid (RNA) sequence (RNA seq) technology to study the global messenger RNA expressional change in the venous samples of five diseased and five control patients. We identified several differentially expressed genes, which were further confirmed by conventional reverse transcription polymerase chain reaction (RT-PCR). Using these significant genes we performed in silico pathway analyses and found distinct transcriptional networks, such as angiogenesis, cell adhesion, vascular injury, and carbohydrate metabolisms that might be involved in the mechanism of VV. Among these significant genes, we also found hyaluronan synthases 2 gene (*HAS2*) played a pivotal role and governed all these pathways. We further confirmed that *HAS2* expression was decreased in the venous samples of patients with VV. Finally, we used a zebrafish model with fluorescence emitting vasculature and red blood cells to see the morphological changes of the venous system and blood flow. We found that *HAS2* knockdown in zebrafish resulted in dilated venous structural with static venous flow. *HAS2* may modulate the transcriptional networks of angiogenesis, cell adhesion, vascular injury, and carbohydrate metabolisms in venous tissues and downregulation of *HAS2* may underlie the mechanism of VV.

## 1. Introduction

Varicose veins (VVs) of lower extremities are among the most easily recognized vascular abnormalities with superficial venous tortuosity and enlargement [1]. Clinical manifestations can vary from leg edema to chronic, disabling venous ulceration and it affects around 25% of the adult population [2]. Age, female sex, family history, pregnancy, obesity, and prolonged standing have been shown to be predisposing factors in several epidemiological studies [3,4,5,6,7,8]. The complex pathophysiology of greater saphenous vein (GSV) valvular reflux and altered extracellular matrix proteins with increased proteolytic enzyme have been addressed as potential mechanisms of VV [9,10,11]. Recently, it has been shown that matrix metalloproteinases may play an important role in the remodeling of venous extracellular matrix and mechanism of VV) [8,10,12,13,14,15]. However, these processes account for only some of the tangled mechanisms, and many more remain to be identified.

Genetic study is a powerful tool to identify the novel mechanism of human diseases. Several candidate genes have been identified as the possible susceptibility genes for VV, such as *COL1A2, FOXC2, NOTCH3, TGF-β,* thrombomodulin, and *VEGF1* [16,17,18,19,20,21,22]. However, whether these genes are true susceptibility genes for VV remains questioned. The study recently provides novel insight into the physiology of lncRNAs and the pathogenesis of varicose veins, but still need to do more for further investigation [2]. Genome-wide association studies (GWASs), a non-candidate-driven approach, investigate the entire genome and can identify unknown or novel disease-associated single nucleotide polymorphisms (SNPs) or genes. [23,24,25,26]. Recently, GWASs have identified several novel genes that may be involved in the mechanism of venous pathogenesis [13,27]. 

However, it is difficult to correlate the identified significant SNPs by GWAS to the mechanism of disease. Therefore, instead of traditional forward genetic approach, in the present study we used a reverse genetic approach. To this end, we first systematically investigated and compared the global expressional changes in the venous samples of diseased and control patients using the next-generation ribonucleic acid (RNA) sequence (RNA seq) technology. We identified several differentially expressed genes that might be involved in the molecular mechanism of VV. We then used a novel zebrafish model to see the phenotypic changes when these genes were perturbed. 

## 2. Methods

### 2.1. Patient Selection

Five patients with VV were consecutively enrolled. All patients were diagnosed and assessed by a vascular surgeon and underwent duplex ultrasonographic scanning to assess suitability for treatment and entry into the study. Inclusion criteria were an age of 18 years or older, the presence of unilateral or bilateral primary symptomatic varicose veins at CEAP classification grade 3 or higher [28] and reflux of GSV of more than 0.5 s on duplex ultrasonography after calf compression [29]. Patients with current deep-vein thrombosis, acute superficial vein thrombosis, bleeding diathesis, pregnancy, and contraindications to the use of general or regional anesthesia were excluded. This study was approved by the institutional review board of the National Taiwan University Hospital and informed consent was obtained from participating subjects.

### 2.2. Surgical Removal of Venous Samples

GSV ablation using the ClosureFAST (VNUS Medical Technologies Inc, San Jose, CA, USA) with concomitant stab phlebectomy were done. All the procedures were performed in a two-day inpatient setting. The GSV was cannulated at the most distal point of venous reflux, and the catheter tip was positioned 2 cm distal to the saphenofemoral junction. The GSV was ablated with the concomitant phlebectomy [30,31]. The specimen collected from the avulsion phlebectomy were used for analysis.

Five control patients were consecutively enrolled. The controls patients had coronary artery disease and ischemic cardiomyopathy. Patients taking steroids or with conditions that could modify leukocyte activity (e.g., rheumatoid arthritis, vasculitis, or other collagen diseases) were excluded. Their GSV samples were harvested during elective coronary artery bypass to serve as venous grafts and the redundant part was used for study. The samples were put into and stored in liquid nitrogen immediately after they were harvested. GSVs competency and diameter were screened by the pre-operative duplex ultrasonography. The baseline characteristics of patients with VV and controls are shown in Table 1. 

### 2.3. Sample Preparation and RNA Sequencing

Total RNA was extracted from the venous samples. The tissue was homogenized with POLYTRON^®^ PT 2100 (Dispergierund Mischtechnik, Littau, Switzerland), and Trizol solution (Gibco BRL, Grand Island, NY, USA) was added for RNA extraction. The extracted RNA was dissolved in diethyl pyrocarbonate-treated distilled water. Spectrophotometry at 260 and 280 nm was performed to measure the amount and quality of RNA. The total RNA samples were first treated with DNase I to degrade any possible deoxyribonucleic acid (DNA) contamination. Then, the messenger RNA (mRNA) was enriched by using the oligo (dT) magnetic beads (for eukaryotes). Mixed with the fragmentation buffer, the mRNA was fragmented into short fragments (about 200 base pair). Then the first strand of complimentary DNA (cDNA) was synthesized by using random hexamer-primer. Buffer, dNTPs, RNase H, and DNA polymerase I were added to synthesize the second strand. The double strand cDNA was purified with magnetic beads. End reparation and 3′-end single nucleotide A (adenine) addition were then performed. Finally, sequencing adaptors were ligated to the fragments. The fragments were enriched by polymerase chain reaction (PCR) amplification. During the quality control step, Agilent 2100 Bioanaylzer (Agilent, Santa Clara, CA, USA) and ABI StepOnePlus Real-Time PCR System (ABI, Foster City, CA 94404, USA) was used to qualify and quantify the sample library. The library products were ready for sequencing via Illumina HiSeqTM 2000 (Illumina, San Diego, CA, USA).

### 2.4. Reverse Transcription Polymerase Chain Reaction

The extraction and quantification of mRNA by means of reverse transcription polymerase chain reaction (RT-PCR) were performed as our standard protocols [32]. The RNA was then converted to complementary DNA by reverse transcription with random hexanucleotides and avian myeloblastosis virus reverse transcriptase (Boehringer, Mannheim, Germany). The primers are as follows: CA4: forward: 5′-CACTGGTGCTACGAGGTTCA-3′, reverse: 5′-GCAACTGTTTGGCCTGGTAT-3′; CHI3L1: forward: 5′-AAGAACAGGAACCCCAACCT-3′, reverse: 5′-TGTCTCTCCGTCCAGGGTAG-3′; FCN1: forward: 5′-GCTCGCTGTCCTGCTAGTCT-3′, reverse: 5′-CTCCAATGACACCTGCCTCT-3′; HAS2: forward: 5′-GCCTCATCTGTGGAGATGGT-3′, reverse: 5′-ATGCACTGAACACACCCAAA-3′. KLK5: forward: 5′-AGTCAGAAAAGGTGCGAGGA-3′, reverse: 5′-TAATCTCCCCAGGACACGAG-3′.

### 2.5. Bioinformatics Analysis of RNA Sequence Data

High quality reads with less than 10% unknown bases and median quality scores above 20 were extracted from the raw RNA-seq data. Cleaned reads were mapped to the reference genome hg19 using SOAPaligner/SOAP2 (Beijing Genomics Institute, Beijing, China) [33] with no more than two mismatches allowed in the alignment. The expression level for each gene was calculated using the RPKM method [34]. If there was more than one transcript for a gene, the longest transcript was used to calculate expression level and coverage. Function annotation could provide gene ontology (GO) annotation information.

### 2.6. Screening of Differentially Expressed Genes (DEGs)

This analysis included the screening of genes that were differentially expressed among samples, GO functional enrichment analysis and KEGG pathway enrichment analysis for these DEGs. We used false discovery rate (FDR) ≤0.001 and the absolute value of log2 ratio ≥1 as the threshold to judge the significance of gene expression difference. 

### 2.7. Pathway and Network Analysis

While so many components are implicated in the mechanism of a complex trait disease, it is difficult to infer which components play a determining role. In this scenario, pathway analysis is a tool used to identify possible biological pathways involving related components. Ingenuity pathway analysis (IPA) is a powerful analysis and pathway-search tool that uncovers the significance of omics data and identifies new targets or candidate biomarkers within the context of biological systems. IPA has been specifically designed for the analysis, integration, and interpretation of data derived from omics experiments. Therefore, in the present study, to derive a possible pathway that may be implicated in the mechanism of AF related thromboembolic stroke, data were analyzed through the use of IPA (QIAGEN Inc., Germantown, MD, USA) [35]. The significance values for the canonical pathways in IPA was calculated using the right-tailed Fisher’s exact test. The *p*-value for a given pathway annotation is calculated by considering (1) the number of DEGs that participate in that pathway and (2) the total number of genes that are known to be associated with that pathway in the selected reference set. Higher the number of DEGs involved, the more likely it is that the association is not due to random chance, and thus more significant the *p*-value is. Similarly, the larger the total number of genes known to be associated with the pathway, the greater the likelihood that an association is due to random chance, and the *p*-value accordingly becomes less significant. The *p*-value identifies statistically significant over-representation of DEGs in a given pathway. The null hypothesis (H_0_) states.

H_0_ = overlap of molecules for a particular biological category is due to chance.

If *p*-value < 0.005, we reject the null hypothesis. 

This implies the association between a set of DEGs in our experiment and a given pathway is not due to random chance. 

### 2.8. Zebrafish Model

*Tg*(*fli1*:*egfp*)*xTg*(*gata1*:*dsRed*) (*egfp*, *enhanced* green fluorescent protein), zebrafish with green fluorescence emitting vasculature and red fluorescence emitting red blood cells were obtained from the Zebrafish International Resource Center (Eugene, OR, USA) [36]. Both wild-type (WT; AB strain) and *Tg*(*fli1*:*egfp*)*xTg*(*gata1*:*dsRed*) zebrafish were kept under a 14-h light and 10-h dark photoperiod at 28.5 °C. The procedures for zebrafish culture, embryo collection, and fluorescence observation have been described previously [37]. We followed the standard criteria to design the zebrafish developmental stages [38]. The zebrafish experiments were approved by the Institutional Animal Care and Use Committee of the National Taiwan University College of Medicine. The anesthesia and euthanasia were performed with diluted tricane methanesulfonate (160 mg/L) and benzocaine hydrochloride (250 mg/L).

### 2.9. Antisense Morpholino Design and Microinjection

Morpholino oligonucleotides (MO) complementary to the 5′- translational start codon flanking sequences of zebrafish targeted gene mRNAs was designed and purchased (Gene Tools LLC, Philomath, OR, USA). Zebrafish *has2*-morpholino (*has2*-MO: 5′-GCTGACCGCTTTATCACATCTCATC-3′) was established based on zebrafish hyaluronan synthase 2 (*has2*) cDNA sequences (Gene Tools LLC, Philomath, OR, USA). It was prepared at stocking concentrations of 1 mM and diluted with double-distilled water to concentrations of 100, 250, 375, and 500 μM (4.5 ng per 2.3 nL). Morpholino oligonucleotide stocks (3 mmol/L) were diluted to 0.3 to 1.2 mmol/L in Danieau solution (58 mmol/L NaCl, 0.7 mmol/L KCl, 0.4 mmol/L MgSO4, 0.6 mmol/L Ca(NO_3_)_2_, 5 mmol/L HEPES, pH 7.6) before injection into the yolks of freshly fertilized fish eggs. Approximately 1 nL of morpholino oligonucleotides were injected into 40 to 180 embryos at the one- to four-cell stage, and the embryos were allowed to develop at 29 °C.

### 2.10. Statistical Methods

Continuous data were presented as means ± standard deviation (SD) and analyzed by using the Mann-Whitney *U* test for two-group comparisons. Categorical data were compared with the Fisher’s exact test. 

## 3. Results

### 3.1. Global Expressional Profiling of VV

Global expressional profiling of venous samples from patients with VV and normal venous samples using the RNA seq technique was performed. The expressional profiling of the significantly up or downregulated genes with log2 ratio >7 were listed in Table 2. Among all these differentially expressed genes, many of them encode proteins functioning as extracellular matrix enzymes, such as Kallikrein-related peptidase 5 (KLK5), chitinase-3-like protein 1 (CHI3L1), hyaluronan synthase 2 (HAS2), and carbonic anhydrase 4 (CA4). These proteins may be involved in remodeling of extracellular matrix proteins and play important roles in vascular integrity and scaffolding. The others are genes encoding cytokines such as chemokine (C-X-C motif) ligand 1 (CXCL1), chemokine (C-X-C motif) ligand 8 (CXCL8), colony stimulating factor 3 (CSF3), and interleukin 6 (IL-6), implicating the importance of paracrine factors related to cell growth and inflammation in the mechanism of VV. 

**Figure 1:**
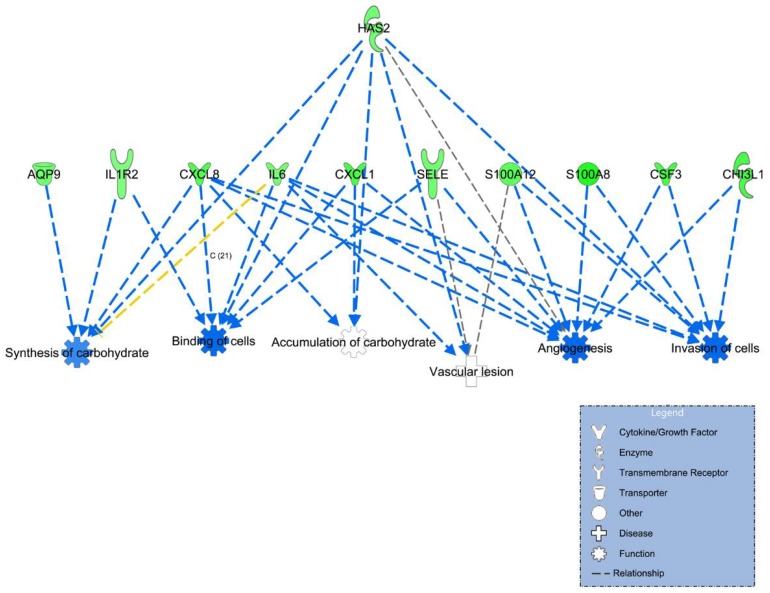
Pathway or network analysis derived from the differentially expressed genes identified by next-generation RNA sequence technology (RNA seq) between venous samples of control patients and those of patients with varicose vein. *N* = 5 for each group.

Because these genes might be implicated in the mechanism of VV, we did a pathway analysis to elucidate the possible translational meaning and significance (Table 3; Figure 1). Interestingly, the results show that decreased angiogenesis, decreased cell-to-cell binding, decreased cell invasion or motility, and enhanced vascular lesion pathways are involved in the mechanism of VV. The results also show that increased carbohydrate accumulation may be involved in the mechanism of VV. Among all these pathways, *HAS2* gene downregulation plays a pivotal role to regulate and govern all these pathways (Figure 1).

**Figure 2:**
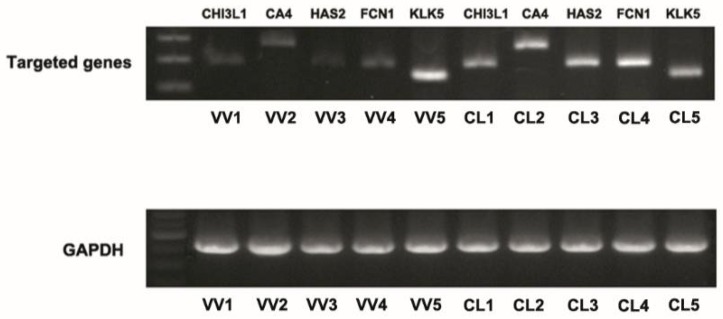
Validation of the differentially expressed genes identified by next-generation RNA sequence technology (RNA seq) using reverse transcription polymerase chain reaction (RT-PCR) in venous samples of control patients (CL) and those of patients with varicose vein (VV). *N* = 5 for each group. *CA4*, carbonic anhydrase 4; *CHI3L1*, chitinase-3-like protein 1; *FCN1*, Ficolin-1; *HAS2*, hyaluronan synthase 2; *KLK*5, kallikrein-related peptidase.

We then validated several differentially expressed genes identified in the RNA seq by conventional RT-PCR using VV and control venous samples. Among all the up or downregulated genes in VV samples, there were several genes encoding enzymes in the extracellular matrix such as KLK5, CHI3L1, HAS2, and CA4. We have validated the expressional changes by conventional RT-PCR for these genes (Figure 2). The result showed decreased mRNA levels of *CHI3L1*, *HAS2*, and *CA4* genes and increased mRNA level of *KLK5* gene in VV samples, which was similar to the result of RNA seq. We also found another gene Ficolin 1 (*FCN1*) encoding extracellular space protein the mRNA level of which was also decreased in the VV samples. 

### 3.2. Downregulation of Hyaluronan Synthases 2 in Venous Tissues from Patients with VV

**Figure 3:**
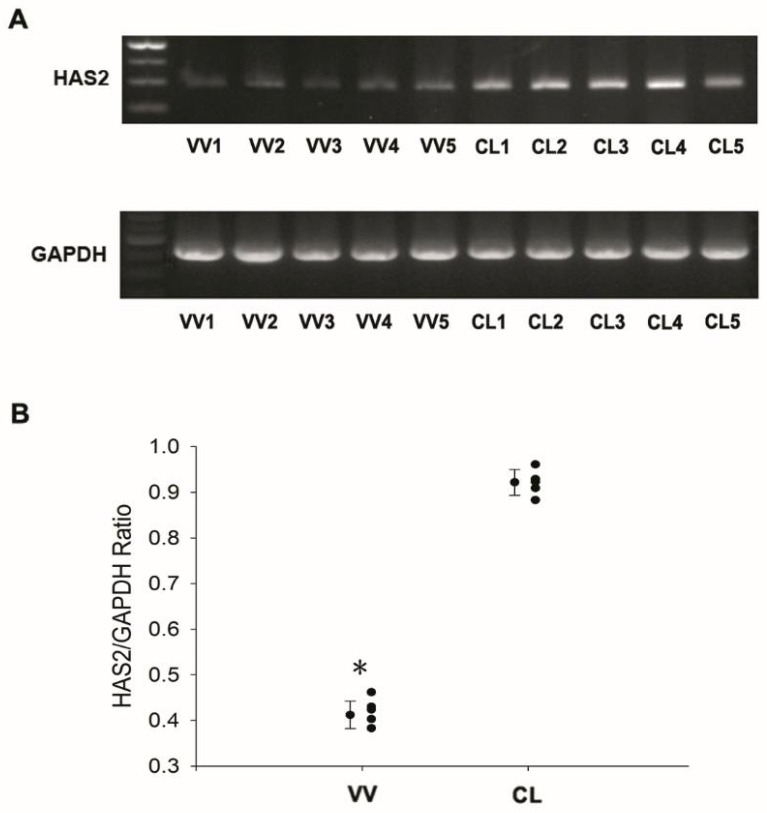
Expression of the hyaluronan synthases 2 gene (*HAS2*) in venous samples from control patients (CL) and patients with varicose vein (VV). (**A**) Expression of *HAS2* gene evaluated by reverse transcription polymerase chain reaction (RT-PCR); (**B**) Dot plot of quantification of RT-PCR product by densitometry. *N* = 5 for each group. * *p* < 0.05.

Among all these extracellular matrix enzyme genes, we were particularly interested in the *HAS2* gene because it played a pivotal role in the pathway analysis, which modulated all the VV-related pathways (Figure 1). The role of HAS in vascular biology has never been addressed before. Because of its possible important role in the mechanism of VV, we repeated the experiment again and confirmed that the mRNA level was significantly lowered in the venous samples from patients with VV (*p* < 0.0001) (Figure 3).

### 3.3. Knockdown of HAS2 Results in Venous Dilation and Blood Flow Stasis in Zebrafish

*Tg*(*fli1*:*egfp*)*xTg*(*gata1*:*dsRed*) zebrafish with green fluorescence emitting vasculature and red fluorescence emitting red blood cells were used to observe the anatomic change of vasculature (green fluorescence) and blood flow (red fluorescence) and to explore the functional role of the identified gene. We first compared the homology of amino acid sequence of *HAS2* among species using Clustal W [39]. *HAS2* is evolutionally conservative and expressed in both low and high vertebrates, indicating its important biological function (Figure S1). The similarity of amino acid sequence of HAS2 between zebrafish and humans was up to 81.3% (Figure S2).

**Figure 4:**
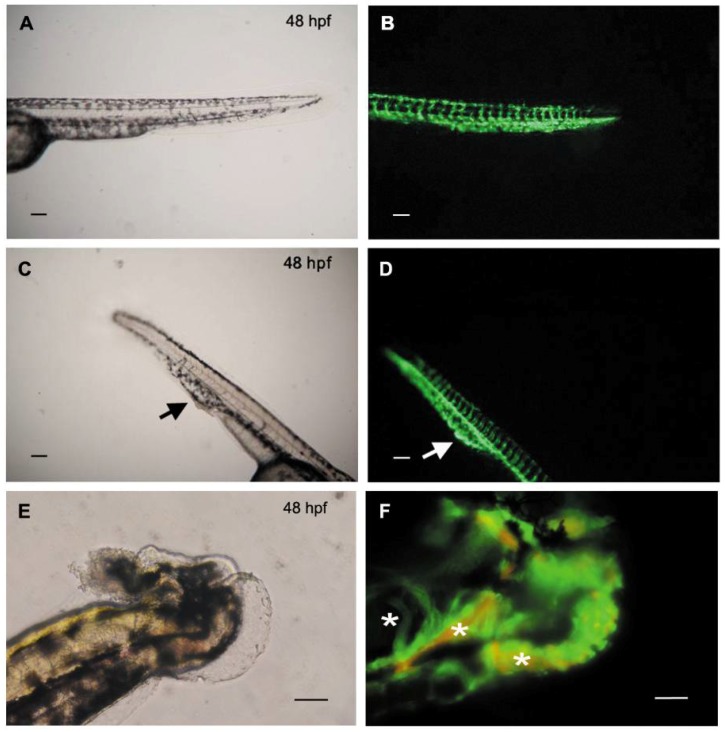
Knockdown of hyaluronan synthases 2 gene (*HAS2*) in zebrafish embryos leads to defective venous structure with blood flow stasis. *Tg*(*fli1*:*egfp*)*xTg*(*gata1*:*dsRed*) zebrafish with green fluorescence emitting vasculature and red fluorescence emitting red blood cells are used to observe the anatomic change of vasculature (green fluorescence) and blood flow (red fluorescence). (**A**) Morphology of the tail of a representative wild-type zebrafish (48 hpf) under bright field microscope; (**B**) Vascular structure of the same zebrafish tail under fluorescence microscope; (**C**) Morphology of the tail of a representative *HAS2*-morpholino (MO) injected zebrafish (48 hpf) under bright field microscope. A protruding bulb in the tail is noted (black arrow), mimicking human phenotype of varicose vein; (**D**) Vascular structure of the same HAS2-MO injected zebrafish tail under fluorescence microscope. A corresponding local dilation of venous structure in the caudal vein plexus (CVP) is found (white arrow); (**E**) Morphology of a representative *HAS2*-morpholino (MO) injected zebrafish (48 hpf) with tail malformation under bright field microscope; (**F**) Vascular structure of the same HAS2-MO injected zebrafish tail under fluorescence microscope. Tangled veins are found in the mal-formatted tail (white stars). Magnification is 100× in (**A**–**D**) and 200× in (**E**,**F**). Scale bar = 100 μm. Hpf indicates hours post-fertilization.

**Figure 5:**
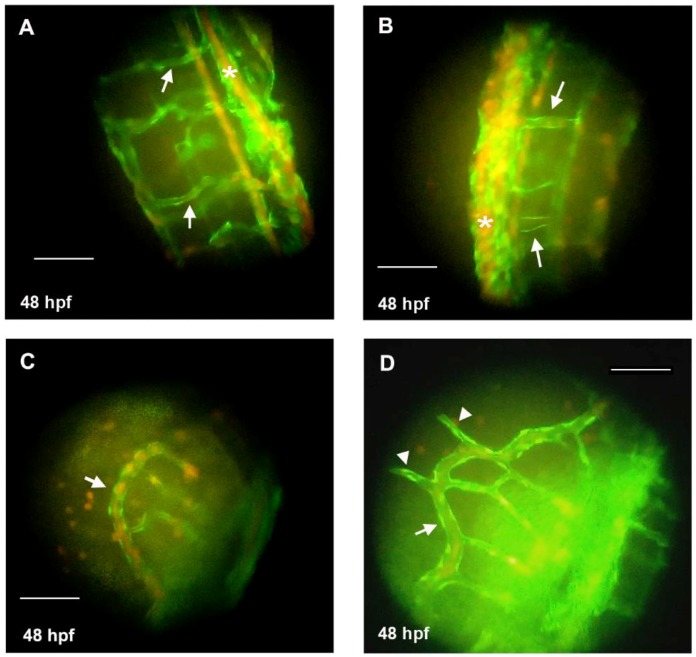
Knockdown of hyaluronan synthases 2 gene (HAS2) in zebrafish embryos leads to dilated venous structure. *Tg*(*fli1*:*egfp*)*xTg*(*gata1*:*dsRed*) zebrafish with green fluorescence emitting vasculature are used to observe the anatomic change of vasculature. (**A**) Inter-segmental veins (ISVs) in the tail of a representative wild-type zebrafish (48 hpf) under fluorescence microscope (arrows); (**B**) Dilated ISVs in the tail of a representative HAS2-morpholino (MO) injected zebrafish (48 hpf) under fluorescence microscope (arrows); (**C**) The sub-intestinal vein (SIV) in the tail of a representative wild-type zebrafish (48 hpf) under fluorescence microscope (arrow); (**D**) The dilated SIV (arrow) with protruding branches (arrowheads) in the tail of a representative HAS2-morpholino (MO) injected zebrafish (48 hpf) under fluorescence microscope. Hpf indicates hours post-fertilization. Magnification is 400×. Scale bar = 100 μm.

Morpholino experiment was quantitatively performed to further define the role of HAS2 in the mechanism of VV. In fish with mild phenotypes, a local dilation of venous structure in the caudal vein plexus (CVP) was found, which resulted in a protruding bulb in the tail, mimicking human VV phenotype of protruding venous dilatation (Figure 4A–D). In fish with moderate phenotypes, dilation of veins, such as intersegmental vein (ISV) or sub-intestinal vein (SIV), was found (Figure 5). Sluggish blood flow in CVP was also noted (Supplementary Movie 1 and Movie 2), mimicking the human VV phenotype of venous blood flow stasis and leg edema. Furthermore, venous branches were also noted in the SIV of the MO-injected fish, which was a consequence of either a dysregulated angiogenetic process or a delay of retraction of leading venous buds during development [40]. It implies that this is a dysregulated angiogenetic process since *HAS2* plays a pivotal role in the angiogenesis network, which is one of the key altered pathways in our transcriptome analysis (Figure 1). In those with severe phenotypes, tangled veins were found in the distal tail which resulted in malformation of tail (Figure 4E,F; Supplementary Movie 3).

Quantitative data after the injection of *HAS2* MO in more than 1000 embryos are shown in Table 4. There was a dose-response relationship. Higher dose of MO (375 uM) was associated with a higher percentage of fish with defective vascular phenotypes than lower dose MO (250 uM). In the highest dose group (500 uM), because of the lethality of high-dose MO, most of the embryos were dead (91%). The survived embryos were probably those without or with mild MO effect and thus there was a relatively lower percentage of survived fish with defect vasculature (43%), compared to those with a medium dose of MO injection (72%) (375 uM) (Table 4). Therefore, we confirm that disruption of HAS2 function directly leads to vascular malformation in vertebrates. 

## 4. Discussion

In the present study, we first used a global expressional profiling to address the molecular mechanism of VV. We further found several important pathways, such as angiogenesis, cell binding, vascular lesion and carbohydrate metabolism possibly involved in the mechanism of VV. We further used a zebrafish model to address the functional role of an identified gene *HAS2* in the mechanism of VV.

In the last decade, the pathophysiological theories of VV shifted from purely mechanical factors to hypotheses focusing on complex molecular and histopathologic alterations in the vessel wall and the extracellular matrix, which may be caused by the genetic inheritance [1,18,19]. In our study, we identified the global expressional change of downregulations of *CHI3L1*, *HAS2*, and *CA4*, and upregulation of *KLK5* in the varicose venous samples with both RNA seq technique and conventional RT-PCR. 

All these genes encode extracellular matrix enzymes, compatible with the concept of histopathologic alterations in the extracellular matrix as a potential mechanism of VV. Extracellular matrix may provide a scaffold for the vascular structure and integrity. When the components of extracellular matrix have been altered (e.g., decomposition of extracellular matrix proteins by excessive enzyme digestion or accumulation of extracellular matrix proteins by decreased enzyme digestion), the scaffold function may deteriorate, resulting in a weakening of vascular wall support and dilatation or protrusion of the vasculature, a pathognomonic picture of VV. Furthermore, after pathway analysis using all the differentially expressed genes, we found decreased angiogenesis, decreased cellular binding and invasion, increased carbohydrate accumulation, and vascular lesion were all involved in the mechanism of VV. All these pathways are novel and have been rarely addressed as the potential mechanism of VV.

Decreased angiogenesis may implicate impaired repair or proliferative activities of vessels and an injury model of VV. Furthermore, an involvement of vascular lesion network also supports the hypothesis of this injury model of VV. Decreased cell-cell binding and invasion may implicate decreased structural integrity of vessels, indicating weakening of vessel wall and possible protrusion of vessel wall, which is the prototype phenotype of VV. Our study also shows that increased carbohydrate accumulation may be involved in the pathogenesis of VV; however, further studies are required to elucidate the detailed mechanism.

As mentioned before, recent studies have also highlighted the pathological changes in the extracellular matrix as the potential mechanism of VV [1,8,10,12,13,15]. Among all these extracellular matrix enzyme genes, *HAS2* expression was decreased in VV samples. Hyaluronan or hyaluronic acid (HA) is a high molecular weight unbranched polysaccharide, and is a major constituent of the extracellular matrix and synthesized by hyaluronan synthases (HAS), which are membrane-bound enzymes and use UDP-α-*N*-acetyl-d-glucosamine and UDP-α-d-glucuronate as substrates to produce the glycosaminoglycan hyaluronan at the cell surface and extrude it through the membrane into the extracellular space. In our zebrafish model, we found disruption of *HAS2* function directly led to venous malformation in vertebrates. Although *HAS2* has been shown to play a role in vascular morphogenesis [41], its specific role in venous tissues and the pathophysiology of the most important human venous diseases—that is, VV—has never been addressed before. The mechanism by which *HAS2* cause VV may be through its pivotal role to modulate and govern multiple pathways that cause VV, such as angiogenesis, cell to cell adhesion, vascular lesion, and carbohydrate metabolism.

There are limitations in the present study. First, there were only five patients in each group and the differences of genetic results we found between VV patients and non-VV patients might be due to something else not related to VV. Second, the genetic changes in the VV samples might be post-transcriptional or happened due to the local environment in the legs, and the zebrafish model only showed a systemic effect. Therefore, HAS2 might not be the gene responsible for VV. Third, we did not have negative scrambled MO control group, positive control group with other MOs targeting to *HAS2* and rescue studies. Therefore, we could not rule out a non-specific effect of MO. Finally, some of our control patients had diabetes that could modify leukocyte activity and might affect the genetic results.

However, there is little doubt that the development and progression of VV and venous ulcer disease is multifactorial and includes a moderate-to-strong genetic component [8]. The genetic studies of VV conducted to date are few and have few functional implications. This is the first study to investigate the global gene expressional profiling in determining the candidate gene of VV. In this study, we show the possible role of *HAS2* gene in the pathogenesis of VV in both global expression and functional study. 

## Figures and Tables

**Table 1:** Clinical characteristics of patients of the two groups

Variables	VV (*n* = 5)	Controls (*n* = 5)
Age (yr)	69.6 ± 14.7	75 ± 11.4
BMI	26.1 ± 2.8	22.6 ± 1.8
Male	2	5
DM	0	3
HTN	2	3
HLP	1	4
Smoke	0	2

BMI, body mass index; DM, diabetes mellitus; HPL, hyperlipidemia; HTN, hypertension; VV, varicose vein; yr, years.

**Table 2:** Highly differentially expressed gene list between venous samples from control (CTL) patients and patients with varicose veins (VV).

Gene	Log2 Ratio	Regulation	Location	Type(s)
	(VV/CTL)	(VV/CTL)		
*CRABP1*	11.98	Up	Cytoplasm	transporter
*SNORA77*	10.16	Up	Other	other
*DPEP1*	9.69	Up	Cytoplasm	peptidase
*PSORS1C1*	9.56	Up	Other	other
*CST2*	9.32	Up	Extracellular Space	other
*HLA-DQA2*	8.68	Up	Plasma Membrane	transmembrane receptor
*CLIC3*	8.46	Up	Nucleus	ion channel
*WFDC10B*	8.20	Up	Extracellular Space	other
*KLK5*	7.77	Up	Extracellular Space	peptidase
*CPNE7*	7.51	Up	Cytoplasm	transporter
*CHAD*	7.39	Up	Extracellular Space	other
*WFDC3*	7.16	Up	Extracellular Space	other
*S100A8*	−12.33	Down	Cytoplasm	other
*GSTT1*	−11.46	Down	Cytoplasm	enzyme
*CXCL1*	−10.01	Down	Extracellular Space	cytokine
*DAB1*	−9.99	Down	Cytoplasm	other
*CHI3L1*	−9.98	Down	Extracellular Space	enzyme
*CSF3*	−9.96	Down	Extracellular Space	cytokine
*CXCL8*	−9.56	Down	Extracellular Space	cytokine
*FCGR3A/FCGR3B*	−9.28	Down	Plasma Membrane	transmembrane receptor
*S100A12*	−8.95	Down	Cytoplasm	other
*CD33*	−8.70	Down	Plasma Membrane	other
*FCN1*	−8.53	Down	Extracellular Space	other
*PQLC2L*	−8.10	Down	Other	other
*AQP9*	−7.93	Down	Plasma Membrane	transporter
*SELE*	−7.85	Down	Plasma Membrane	transmembrane receptor
*HAS2*	−7.69	Down	extracellular matrix	enzyme
*CA4*	−7.63	Down	Plasma Membrane	enzyme
*IL6*	−7.62	Down	Extracellular Space	cytokine
*THEM5*	−7.58	Down	Cytoplasm	enzyme
*ZNF385B*	−7.52	Down	Nucleus	other
*SRGAP2B*	−7.39	Down	Other	other
*FCGR2C*	−7.18	Down	Plasma Membrane	transmembrane receptor
*IL1R2*	−7.13	Down	Plasma Membrane	transmembrane receptor
*ALAS2*	−7.08	Down	Cytoplasm	enzyme
*HHATL*	−7.08	Down	Cytoplasm	enzyme

**Table 3:** Pathways involved in the transcriptional regulation of varicose veins

Diseases or Functions Annotation	*p*-Value *	Predicted Activation State	Molecules
Angiogenesis	8.48 × 10^−5^	Decreased	CHI3L1, CSF3, CXCL1, CXCL8, HAS2, IL6, S100A12, S100A8, SELE
Binding of cells	3.07 × 10^−4^	Decreased	CXCL1, CXCL8, HAS2, IL1R2, IL6, SELE
Invasion of cells	3.43 × 10^−4^	Decreased	CHI3L1, CSF3, CXCL1, CXCL8, HAS2, IL6, S100A12, S100A8
Vascular lesion	7.74 × 10^−4^		HAS2, IL6, S100A12, SELE
Synthesis of carbohydrate	1.32 × 10^−3^		AQP9, CXCL8, HAS2, IL1R2, IL6
Accumulation of carbohydrate	1.93 × 10^−3^		CXCL1, CXCL8, HAS2

* *p*-value was calculated using the right-tailed Fisher’s exact test.

**Table 4:** Morphological phenotypes of zebrafish embryos (fli1/gata1) derived from fertilized eggs injected with *HAS2*-MO.

Injection Dose (μM)	Injected Embryos	Survival Embryos ^1^	GFP-Positive Embryos	Embryos without Defective Vascular Phenotypes (%) *	Embryos with Defective Vascular Phenotypes (%) *
		(Survival Rates %) ^2^			
250	231	145 (62%)	132	84 (63%)	48 (37%)
375	563	254 (45%)	240	68 (28%)	172 (72%)
500	502	49 (9%)	28	16 (57%)	12 (43%)

^1^ Total numbers of the survival embryos at 48 hpf. ^2^ Survival rates indicate the numbers of survival embryos among the numbers of injected embryos at 48 hpf. * Percentages indicate the numbers of no defect or vascular defective embryos among the numbers of GFP-positive embryos at 48 hpf. hpf, hours post-fertilization. GFP, green fluorescent protein.

## Supplementary Materials

The Figure S1, Figure S2 and Supplementary Movie 1–3 are available online at http://www.mdpi.com/2077-0383/7/12/537/s1.
