# Supplementary material for: Global Expression Profiling Identifies a Novel Hyaluronan Synthases 2 Gene in the Pathogenesis of Lower Extremity Varicose Veins
Source: J Clin Med. 2018 Dec 11;7(12):537. doi: 10.3390/jcm7120537 (PMC6306753; doi:10.3390/jcm7120537)
Supplement: Supplementary file 1 [file jcm-07-00537-s001.zip › jcm-385776-supplementary/supplementary figures.docx]

Supplementary Data

Global Expression Profiling Identifies a Novel Hyaluronan Synthases 2 Gene in the Pathogenesis of Lower Extremity Varicose Veins

Chia-Shan Hsieh ^1,2^, Chia-Ti Tsai ^3,4^, Yau-Hung Chen ^5^, Sheng-Nan Chang ^6^, Juey-Jen Hwang ^3,6^, Eric Y. Chuang ^1,2,^*, and I-Hui Wu ^4,7,^*

^1^ Department of Life Science, Genome and Systems Biology Degree Program, National Taiwan University, Taipei, 10617, Taiwan; cometrise@gmail.com

^2^ Bioinformatics and Biostatistics Core, Center of Genomic Medicine, National Taiwan University, Taipei, 10055, Taiwan

^3^ Division of Cardiology, Department of Internal Medicine, National Taiwan University College of Medicine and Hospital, Taipei, 10002, Taiwan; cttsai1999@gmail.com (C.-T.T.); jueyhwang@ntu.edu.tw (J.-J.H.)

^4^ Graduate Institute of Clinical Medicine, College of Medicine, National Taiwan University, Taipei, 10051, Taiwan

^5^ Department of Chemistry, Tamkang University, Taipei, 25137, Taiwan; yauhung@mail.tku.edu.tw

^6^ Department of Internal Medicine, National Taiwan University Hospital Yun-Lin Branch, Yun-Lin, 64041, Taiwan; p95421008@ntu.edu.tw

^7^ Department of Surgery, National Taiwan University Hospital, Taipei, 10002, Taiwan

***** Correspondence: chuangey@ntu.edu.tw (E.Y.C.); Tel: +886-2-3366-3660; aaronihuiwu@gmail.com (I.-H.W.); Tel.: +886-2-23123456 (ext. 65735) (E.Y.C. & I.-H.W.)**Supplementary Figure 1.** Amino acid sequence of hyaluronan synthases 2 (*HAS2*) of various vertebrate species using CLUSTAL 2.1 multiple sequence alignment.

| **Gene names**  **(species)** | **Coding region (aa)** | **Mw (kDa)** | **pI** | **GenBank accession number** | **References** |
| --- | --- | --- | --- | --- | --- |
| *has2* |  |  |  |  |  |
| *H.sapiens* | 552 | 63.57 | 8.85 | NP_005319 | Stein et al., 2014 |
| *M.musculus* | 552 | 63.51 | 8.85 | NP_032242 | Cai et al., 2013 |
| *R.norvegicus* | 552 | 63.53 | 8.85 | AAB63209 | Sakaguchi and Midura, 1997 |
| *B.taurus* | 552 | 63.46 | 8.9 | NP_776504 | Zimin et al., 2009 |
| *G. gallus* | 552 | 63.74 | 8.75 | NP_990137 | Rada et al., 2010 |
| *X. laevis* | 551 | 63.69 | 8.79 | NP_001083837 | Ori et al., 2006 |
| *D.rerio* | 552 | 63.42 | 8.96 | NP_705936 | Bonetti et al., 2014 |

*M.musculus* MHCERFLCVLRIIGTTLFGVSLLLGITAAYIVGYQFIQTDNYYFSFGLYGAFLASHLIIQ 60

*R.norvegicus*  MHCERFLCVLRIIGTTLFGVSLLLGITAAYIVGYQFIQTDNYYFSFGLYGAFLASHLIIQ 60

*H.sapiens* MHCERFLCILRIIGTTLFGVSLLLGITAAYIVGYQFIQTDNYYFSFGLYGAFLASHLIIQ 60

*B.taurus*  MHCERFLCILRIIGTTLFGVSLLLGITAAYIVGYQFIQTDNYYFSFGLYGAFLASHLIIQ 60

*G.gallus* MYCERFICILRILGTTLFGVSLLLGITAAYIVGYQFIQTDNYYFSFGLYGAILASHLIIQ 60

*X.laevis*  MHCERFICILRIIGTTLFGVSLLLGISAAYIVGYQFIQTDNYYFSFGLYGAILALHLIIQ 60

*D.rerio* MRCDKAVSYLRIVGTTLFGISLLVGISTAYIMGYKLVTTPGNYLSFGLYGAILVIHLIIQ 60

*M.musculus* SLFAFLEHRKMKKSLETPIKLNKTVALCIAAYQEDPDYLRKCLQSVKRLTYPGIKVVMVI 120

*R.norvegicus* SLFAFLEHRKMKKSLETPIKLNKTVALCIAAYQEDPDYLRKCLQSVKRLTYPGIKVVMVI 120

*H.sapiens* SLFAFLEHRKMKKSLETPIKLNKTVALCIAAYQEDPDYLRKCLQSVKRLTYPGIKVVMVI 120

*B.taurus*  SLFAFLEHRKMKKSLETPIKLNKTVALCIAAYQEDPDYLRKCLQSVKRLTYPGIKVVMVI 120

*G.gallus* SLFAYLEHRKMKRSLETPIKLNKTVALCIAAYQEDPDYLRKCLLSVKRLTYPGIKVVMVI 120

*X.laevis*  SLFAFLEHRKMKRSLETPIKLNKSVALCIAAYQEDEDYLRKCLLSVKRLTYPGMKVIMVI 120

*D.rerio* SVFALLEHRNMKRSLETPIKLNKSLALCIAAYQEDPNYLRKCLISVKRLTYPGIKVIMVI 120

*M.musculus*  DGNSDDDLYMMDIFSEVMGRDKSATYIWKNNFHEKGPGETEESHKESSQHVTQLVLSNKS 180

*R.norvegicus* DGNSDDDLYMMDIFSEVMGRDKSVTYIWKNNFHERGPGETEESHKESSQHVTQLVLSNKS 180

*H.sapiens*  DGNSEDDLYMMDIFSEVMGRDKSATYIWKNNFHEKGPGETDESHKESSQHVTQLVLSNKS 180

*B.taurus* DGNSEDDLYMMDIFSEVMGRDKSATYIWKNNYHVKGPGETDESHKESSQHVTQLVLSNKS 180

*G.gallus* DGNSEDDVYMMDIFTEIMGRDKSATYIWSNNFHDKGPGETEESHRESMQHVSQLVLSNKS 180

*X.laevis* DGNSDDDLYMMNIFREIMGNDSCATYVWKNNFHMKGPNETDETHRESMQHVTQMVLSNRN 180

*D.rerio* DGNNDDDCYMMEIFREIMGRDKAATYIWKSNYHHRGPEETEESYATSLQHVSHLVLNNKC 180

*M.musculus* ICIMQKWGGKREVMYTAFRALGRSVDYVQVCDSDTMLDPASSVEMVKVLEEDPMVGGVGG 240

*R.norvegicus* ICIMQKWGGKREVMYTAFRALGRSVDYVQVCDSDTMLDPASSVEMVKVLEEDPMVGGVGG 240

*H.sapiens* ICIMQKWGGKREVMYTAFRALGRSVDYVQVCDSDTMLDPASSVEMVKVLEEDPMVGGVGG 240

*B.taurus* ICTMQKWGGKREVMYTAFRALGRSVDYVQVCDSDTMLDPASSVEMVKVLEEDPMVGGVGG 240

*G.gallus*  VCIMQKWGGKREVMYTAFKALGEAWNYVQVCDSDTMLDPASSVEMVKVLEEDPMVGGVGG 240

*X.laevis* VCIMQKWNGKREVMYTAFKALGRSVDYVQVCDSDTVLDPASSVEMVKVLEEDIMVGGVGG 240

*D.rerio* VCIMQKWGGKREVMYTAFKALGRSVDYVQVCDSDTMLDPASSVEMVKVLEEDPNVGGVGG 240

*M.musculus* DVQILNKYDSWISFLSSVRYWMAFNIERACQSYFGCVQCISGPLGMYRNSLLHEFVEDWY 300

*R.norvegicus* DVQILNKYDSWISFLSSVRYWMAFNIERACQSYFGCVQCISGPLGMYRNSLLHEFVEDWY 300

*H.sapiens* DVQILNKYDSWISFLSSVRYWMAFNIERACQSYFGCVQCISGPLGMYRNSLLHEFVEDWY 300

*B.taurus* DVQILNKYDSWISFLSSVRYWMAFNIERACQSYFGCVQCISGPLGMYRNSLLHEFVEDWY 300

*G.gallus* DVQILNKYDSWISFLSSVRYWMAFNIERACQSYFGCVQCISGPLGMYRNSLLHEFVEDWY 300

*X.laevis* DVQILNKYDSWISFLSSVRYWMAFNIERACQSYFGCVQCISGPLGMYRNSLLHEFIEDWY 300

*D.rerio* DVQILNKYESWVSFLSSVRYWMAFNIERACQSYFGCVQCISGPLGMYRNSLLHEFLEDWY 300

*M.musculus* NQEFMGNQCSFGDDRHLTNRVLSLGYATKYTARSKCLTETPIEYLRWLNQQTRWSKSYFR 360

*R.norvegicus* NQEFMGNQCSFGDDRHLTNRVLSLGYATKYTARSKCLTETPIEYLRWLNQQTRWSKSYFR 360

*H.sapiens* NQEFMGNQCSFGDDRHLTNRVLSLGYATKYTARSKCLTETPIEYLRWLNQQTRWSKSYFR 360

*B.taurus* NQEFMGSQCSFGDDRHLTNRVLSLGYATKYTARSKCLTETPIEYLRWLNQQTRWSKSYFR 360

*G.gallus* NQEFMGSQCSFGDDRHLTNRVLSLGYATKYTARSKCLTETPIEYLRWLNQQTRWSKSYFR 360

*X.laevis* NQEFLGSQCSFGDDRHLTNRVLSLGYATKYTARSKCLTETPTEYLRWLNQQTRWSKSYFR 360

*D.rerio* DQTFMGSHCSFGDDRHLTNRVLSLGYATKYTARSKCLTETPITYLRWLNQQTRWSKSYFR 360

*M.musculus* EWLYNAMWFHKHHLWMTYEAVITGFFPFFLIATVIQLFYRGKIWNILLFLLTVQLVGLIK 420

*R.norvegicus* EWLYNAMWFHKHHLWMTYEAVITGFFPFFLIATVIQLFYRGKIWNILLFLLTVQLVGLIK 420

*H.sapiens*  EWLYNAMWFHKHHLWMTYEAIITGFFPFFLIATVIQLFYRGKIWNILLFLLTVQLVGLIK 420

*B.taurus* EWLYNAMWFHKHHLWMTYEAVITGFFPFFLIATVIQLFYRGKIWNTLLFLLTVQLVGLIK 420

*G.gallus* EWLYNAMWFHKHHLWMTYEAVITGFFPFFLIATVIQLFYRGKIWNILLFLLTVQLVGLIK 420

*X.laevis* EWLYNSLWFHKHHLWMTYEAVITGFFPFFLIATVIQLFYRGRIWNILLFLLTVQLVGLIK 420

*D.rerio*  EWLYNSLWFHKHHLWMTYEAVITGFFPFFLIATAIQLFYQGRIWNILLFLLIVQVVALIK 420

*M.musculus* SSFASCLRGNIVMVFMSLYSVLYMSSLLPAKMFAIATINKAGWGTSGRKTIVVNFIGLIP 480

*R.norvegicus* SSFASCLRGNIVMVFMSLYSVLYMSSLLPAKMFAIATINKAGWGTSGRKTIVVNFIGLIP 480

*H.sapiens* SSFASCLRGNIVMVFMSLYSVLYMSSLLPAKMFAIATINKAGWGTSGRKTIVVNFIGLIP 480

*B.taurus* SSFASCLRGNIVMVFMSLYSVLYMSSLLPAKMFAIATINKAGWGTSGRKTIVVNFIGLIP 480

*G.gallus* SSFASFLRGNIVMVFMSLYSVLYMSSLLPAKMFAIATINKAGWGTSGRKTIVVNFIGLIP 480

*X.laevis*  SSFASALRGNIVMVFMSFYSVLYMSSLLPAKMFAIATINKAGWGTSGRKTIVVNFIGLIP 480

*D.rerio*  SSFASCLRGNIVMVFMSFYSVLYMSSLLPAKMFAIATINKSGWGTSGRKTVVVNFIGLIP 480

*M.musculus* VSVWFTILLGGVIFTIYKESKKPFSESKQTVLIVGTLIYACYWVMLLTLYVVLINKCGRR 540

*R.norvegicus* VSVWFTILLGGVIFTIYKESKKPFSESKQTVLIVGTLIYACYWVVLLTLYVVLINKCGRR 540

*H.sapiens*  VSVWFTILLGGVIFTIYKESKRPFSESKQTVLIVGTLLYACYWVMLLTLYVVLINKCGRR 540

*B.taurus* VSVWFTILLGGVIFTIYKESKKPFSESKQTVLIVGTLLYACYWVMLLTLYVVLINKCGRR 540

*G.gallus* VSIWFTILLGRVIFTIYKESKKPFSESKTTVLVIGTILYACYWVLLLTLYLVLITKCGRR 540

*X.laevis* ITVWFTILLGGVCYTIWRETKKPFSESEKIVLAVGAILYACYWVMLLTMYVSLVMKCGRR 540

*D.rerio* ISIWFTILFVGIIYTIIQETRKPFPESEKVVLIIGAIVYISYWVVFLTLYAVLIMKCGKR 540

*M.musculus*  KKGQQYDMVLDV 552

*R.norvegicus* KKGQQYDMVLDV 552

*H.sapiens*  KKGQQYDMVLDV 552

*B.taurus* KKGQQYDMVLDV 552

*G.gallus* KKEQHYDMVLDV 552

*X.laevis*  RKEPQHDLVLA- 551

*D.rerio* KKGQQYDMVLDV 552

**Supplementary Figure 2.** Similarity matrix of hyaluronan synthases 2 amino acid sequence among difference vertebrate species and phylogenetic tree based on sequence hyaluronan synthases 2 amino acid sequence.

|  | ***H.sapiens*** | ***M.musculus*** | ***R.norvegicus*** | ***G. gallus*** | ***B.taurus*** | ***X.laevis*** | ***D.rerio*** |
| --- | --- | --- | --- | --- | --- | --- | --- |
| *H.sapiens* | 100 | 98.91 | 98.37 | 93.3 | 98.73 | 88.2 | 81.34 |
| *M.musculus* |  | 100 | 99.46 | 93.3 | 98.37 | 88.2 | 82.07 |
| *R.norvegicus* |  |  | 100 | 92.93 | 97.83 | 87.66 | 82.25 |
| *G. gallus* |  |  |  | 100 | 93.3 | 87.66 | 81.88 |
| *B.tauru* |  |  |  |  | 100 | 88.2 | 81.7 |
| *X. laevis* |  |  |  |  |  | 100 | 81.13 |
| *D.rerio* |  |  |  |  |  |  | 100 |

**
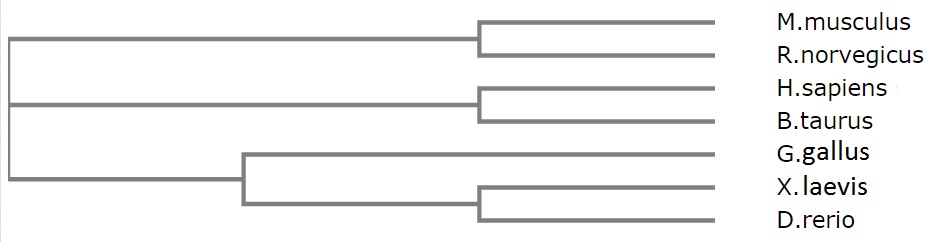
**

**Supplementary Movie 1.** Blood flow in the posterior cardinal vein in one of the representative control fish. The venous flow in the intersegmental vein returns into the posterior cardinal vein smoothly. The posterior cardinal vein flow is also smooth and rapid.

**Supplementary Movie 2.** Blood flow in the posterior cardinal vein in one of the representative hyaluronan synthases 2 gene knockdown fish. The venous flow is sluggish when entering from the intersegmental vein to the posterior cardinal vein. The posterior cardinal vein flow is also slow and sluggish.

**Supplementary Movie 3.** The venous structure and blood flow in a representative hyaluronan synthases 2 gene knockdown fish with tail enlargement and malformation. The veins in the tail are tangled and the blood flow is sluggish.
